# Suppressed N fixation and diazotrophs after four decades of fertilization

**DOI:** 10.1186/s40168-019-0757-8

**Published:** 2019-10-31

**Authors:** Kunkun Fan, Manuel Delgado-Baquerizo, Xisheng Guo, Daozhong Wang, Yanying Wu, Mo Zhu, Wei Yu, Huaiying Yao, Yong-guan Zhu, Haiyan Chu

**Affiliations:** 1 State Key Laboratory of Soil and Sustainable Agriculture, Institute of Soil Science, Chinese Academy of Sciences, 71 East Beijing Road, Nanjing, 210008 China; 2 University of Chinese Academy of Sciences, Beijing, 100049 China; 3 Cooperative Institute for Research in Environmental Sciences, University of Colorado, Boulder, CO 80309 USA; 4 Departamento de Biología y Geología, Física y Química Inorgánica, Escuela Superior de Ciencias Experimentales y Tecnología, Universidad Rey Juan Carlos, c/ Tulipán s/n, 28933 Móstoles, Spain; 5 Institute of Soil and Fertilizer Research, Anhui Academy of Agricultural Sciences, 40 South Nongke Road, Hefei, 230031 China; 6 High School Affiliated to Nanjing Normal University, Nanjing, 210003 China; 7 Key Laboratory of Urban Environment and Health, Institute of Urban Environment, Chinese Academy of Sciences, Xiamen, 361021 China; 8 School of Environmental Ecology and Biological Engineering, Wuhan Institute of Technology, Wuhan, 430205 China

**Keywords:** Diazotrophs, Nitrogen fixation rates, Ecological clusters, Long-term fertilization

## Abstract

**Background:**

N fixation is one of the most important microbially driven ecosystem processes on Earth, allowing N to enter the soil from the atmosphere, and regulating plant productivity. A question that remains to be answered is whether such a fundamental process would still be that important in an over-fertilized world, as the long-term effects of fertilization on N fixation and associated diazotrophic communities remain to be tested. Here, we used a 35-year fertilization experiment, and investigated the changes in N fixation rates and the diazotrophic community in response to long-term inorganic and organic fertilization.

**Results:**

It was found that N fixation was drastically reduced (dropped by 50%) after almost four decades of fertilization. Our results further indicated that functionality losses were associated with reductions in the relative abundance of keystone and phylogenetically clustered N fixers such as *Geobacter* spp.

**Conclusions:**

Our work suggests that long-term fertilization might have selected against N fixation and specific groups of N fixers. Our study provides solid evidence that N fixation and certain groups of diazotrophic taxa will be largely suppressed in a more and more fertilized world, with implications for soil biodiversity and ecosystem functions.

## Background

Biological nitrogen (N) fixation is one of the most important ecological processes on Earth, and is responsible for the fixation of up to 100 Tg N year^−1^ from the atmosphere globally [1–3]. However, N fixation and its associated microbial communities have been largely challenged by the industrial Haber process, and later by inorganic and organic fertilization [4], which provides 32 Tg N year^−1^ to global croplands [5]. Such a large amount of fertilization might relegate N fixers to a second place [6, 7] and could have long-term consequences for these important microbial communities and ecosystem processes in the future [8, 9]. Surprisingly, relatively little is known about the long-term effects of inorganic and organic fertilization on N fixation rates and their associated N fixers in terrestrial ecosystems.

Short-term additions of N fertilizers can result in an increase in the abundance of fast-growing diazotrophs [10]. These microbial communities may use resources from fertilizers to support their own vegetative growth, instead of fixing nitrogen [11], which is known to be an energy-expensive process [12]. Much less is known, however, about the long-term consequences (over decades) of soil fertilization (e.g., N fertilizer additions) on N fixation rates and their associated diazotrophic communities. We posit that, in fertilized environments, N fixation and fixers will become less and less important as time passes. However, experimental evidence supporting this hypothesis is lacking. Following natural selection theories [13–15], we hypothesized that fertilization should suppress N fixation and drastically change the community composition of N fixers, which may no longer be needed to fix N_2_ from the atmosphere. Fertilization could be particularly detrimental for oligotrophic microbial communities and for obligate N fixers that have limited ability to downregulate fixation. However, fertilization could benefit copiotrophic and facultative N fixers that are capable of downregulating fixation, such as *Bradyrhizobium* spp. [16, 17].

Here, we used soils from a 35-year fertilization experiment and the most advanced sequencing technology to target *nifH* genes that encode the reductase subunit of nitrogenase [18]. The role of fertilization in regulating N fixation and the phylogeny and community composition of N fixers were evaluated [19] by using contrasting fertilization management strategies: non-fertilization (control), chemical fertilization (NPK), chemical fertilization with wheat straw (NPK + WS), chemical fertilization with pig manure (NPK + PM), and chemical fertilization with cow manure (NPK + CM).

## Results

### N fixation and N fixers under long-term fertilization scenarios

**Fig. 1:**
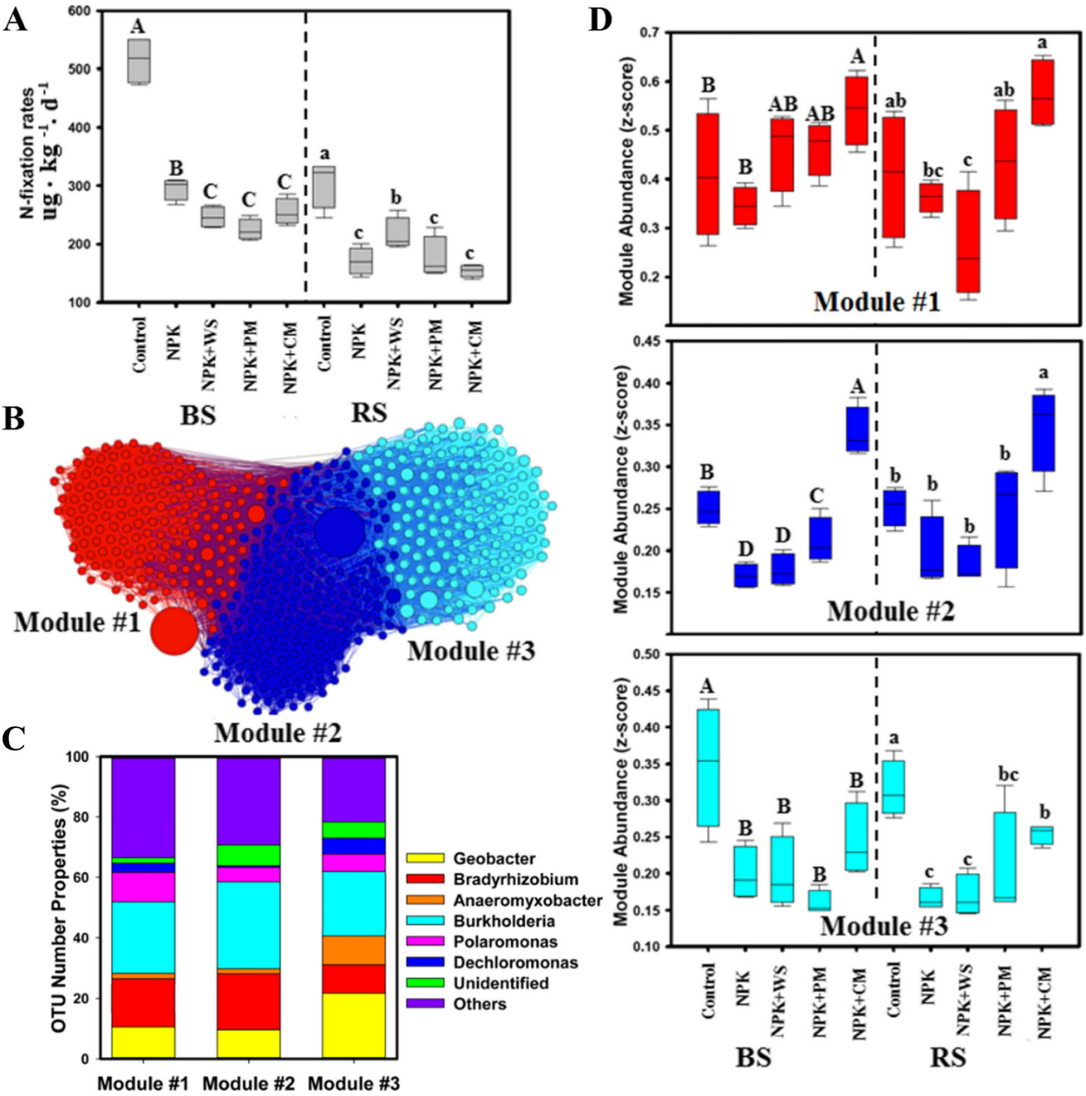
**a** Nitrogen fixation rates in different fertilization treatments. **b** Network diagram with nodes colored according to each of the three main ecological clusters (modules #1–3). **c** Operational taxonomic unit (OTU) number properties of the dominant diazotrophic genera in the three main ecological clusters. **d** Relative abundance of the ecological clusters in different fertilization treatments. Different letters indicate the values that differ significantly among treatments at *P* < 0.05 (Duncan’s test) in bulk soil (A, B, C) and rhizosphere soil (a, b, c). BS: bulk soil, RS: rhizosphere soil. Fertilization treatments: control, non-fertilization; NPK fertilization, NPK (urea, superphosphate, and potassium chloride); NPK + WS, NPK with wheat straw; NPK + PM, NPK with pig manure; NPK + CM, NPK with cow manure (NPK + CM)

**Fig. 2:**
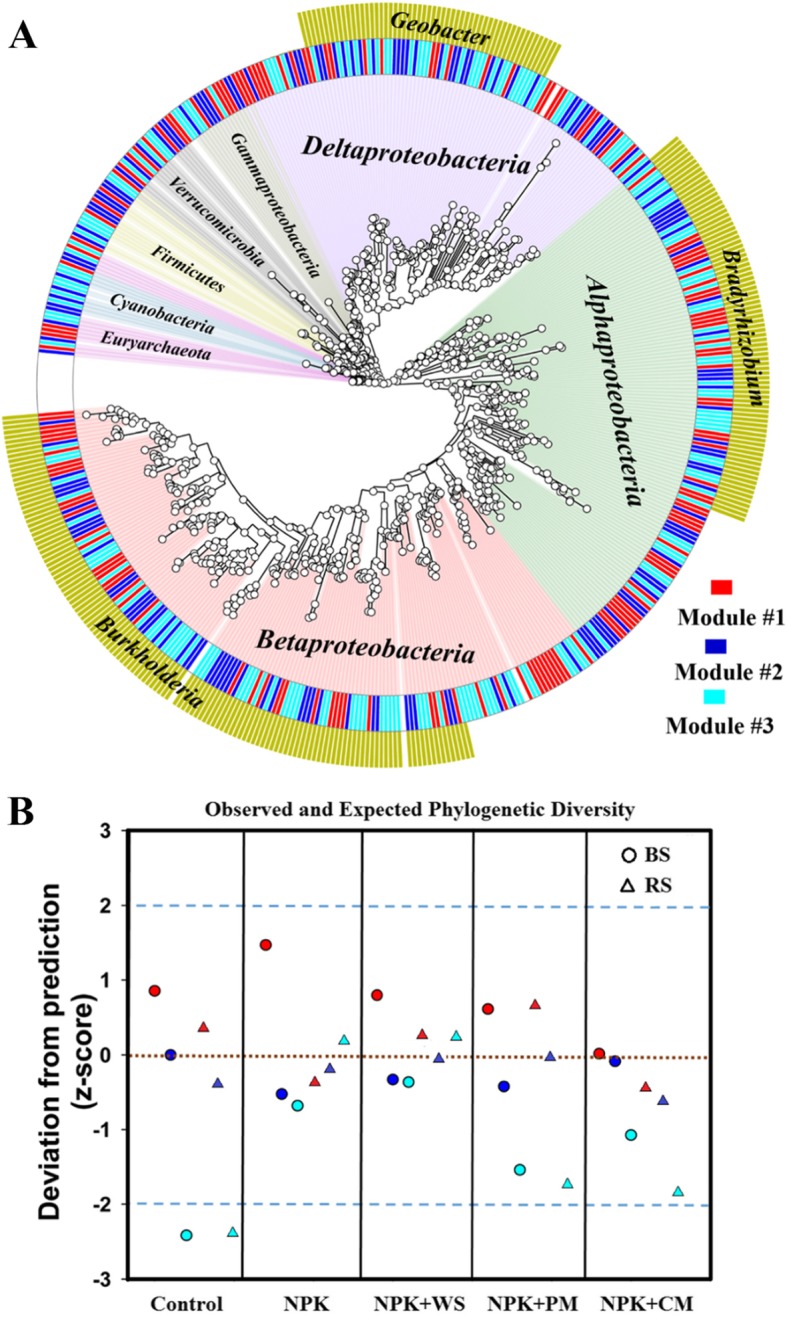
**a** Phylogenetic tree displaying the taxonomic information on dominant soil diazotrophic phylotypes in three main ecological clusters (modules #1–3). **b** The standardized difference, in units of standard deviation (z-score), between observed and expected phylogenetic diversity assuming random sampling for each module. The dotted brown line represents the expected phylogenetic diversity for each treatment and the blue dashed lines represent the 95% confidence intervals. The points either above (2) or below (− 2) the blue lines represent the degree to which those ecological clusters are phylogenetically over-dispersed or clustered, respectively. Red, module #1; blue, module #2; cyan, module #3. BS: bulk soil, RS: rhizosphere soil. Fertilization treatments: control, non-fertilization; NPK fertilization, NPK (urea, superphosphate, and potassium chloride); NPK + WS, NPK with wheat straw; NPK + PM, NPK with pig manure; NPK + CM, NPK with cow manure (NPK + CM)

Our results indicated that N fixation rates were significantly suppressed by a wide range of fertilizers after almost four decades of fertilization (Fig. 1a). We found that N fixation rates dropped by 50%, which was more noticeable in bulk soils than in the rhizosphere (Additional file 1: Table S6). We then evaluated the effects of long-term fertilization on N fixers. To do so, we built a correlation network incorporating the detected dominant diazotrophic phylotypes and found three ecological clusters of N fixers strongly co-occurring with each other (modules #1, #2, and #3; Fig. 1b). Each ecological cluster consisted of multiple diazotrophic species attributing to different genera (Fig. 1c; Fig. 2a). *Bradyrhizobium* and *Burkholderia* were the most dominant genera of N fixers in module #1 and module #2; *Geobacter* and *Anaeromyxobacter* dominated module #3 (Fig. 1c). Long-term fertilization resulted in drastic changes in the relative abundance of ecological clusters; the relative abundance of module #3 was strongly reduced and that of modules #1 and #2 was somewhat increased—particularly under NPK + CM additions (Fig. 1d). Similar results were found for bulk and rhizosphere soil (Additional file 1: Table S8).

We also constructed a phylogenetic tree with the dominant diazotrophic phylotypes, and found that the N fixers of the *Geobacter* genus were highly clustered in module #3. On the contrary, N fixers within the dominant genera *Bradyrhizobium* and *Burkholderia* were randomly distributed in module #1 and module #2 (Fig. 2a). We then calculated the phylogenetic diversity of each ecological cluster and further compared these observed values with the expected random values for each cluster [20]. We found that the observed phylogenetic diversity for module #1 and module #2 was consistent with the random predictions (within the 95% confidence interval) across different fertilization treatments (Fig. 2b). However, the observed phylogenetic diversity for module #3 deviated significantly below the random prediction under long-term non-fertilization scenarios; this is indicative of phylogenetic clustering. Meanwhile, under long-term application of fertilizers, the trends for module #3 were indicative of phylogenetic randomness (Fig. 2b). These results suggest that long-term fertilization may have selected against the N fixers associated with module #3 (mostly *Geobacter* spp).

### Linking N fixers to N fixation under long-term fertilization scenarios

**Fig. 3:**
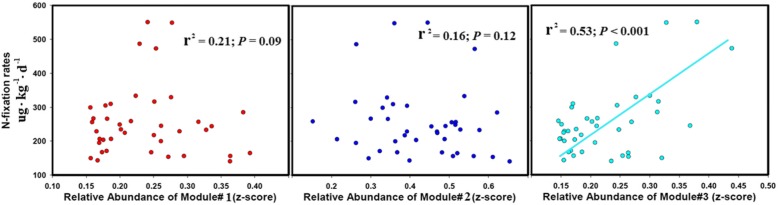
Regressions between the nitrogen fixation rates and the relative abundance of the main diazotrophic ecological clusters. From left to right are modules #1, #2, and #3, represented by red, blue, and cyan plots, respectively

A strong and significant positive association between the relative abundance of module #3 and N fixation rates was found (Fig. 3). However, no significant association was detected between the relative abundance of module #1, module #2, and N fixation rates (Fig. 3). Fifty diazotrophic phylotypes were detected to be highly positively correlated with nitrogen fixation rates using random forest regression (Additional file 2: Figure S2 and S3). These phylotypes were mostly included in module #3 (20/50) when compared to module #1 (3/50) and module #2 (4/50) (Additional file 1: Table S10).

**Fig. 4:**
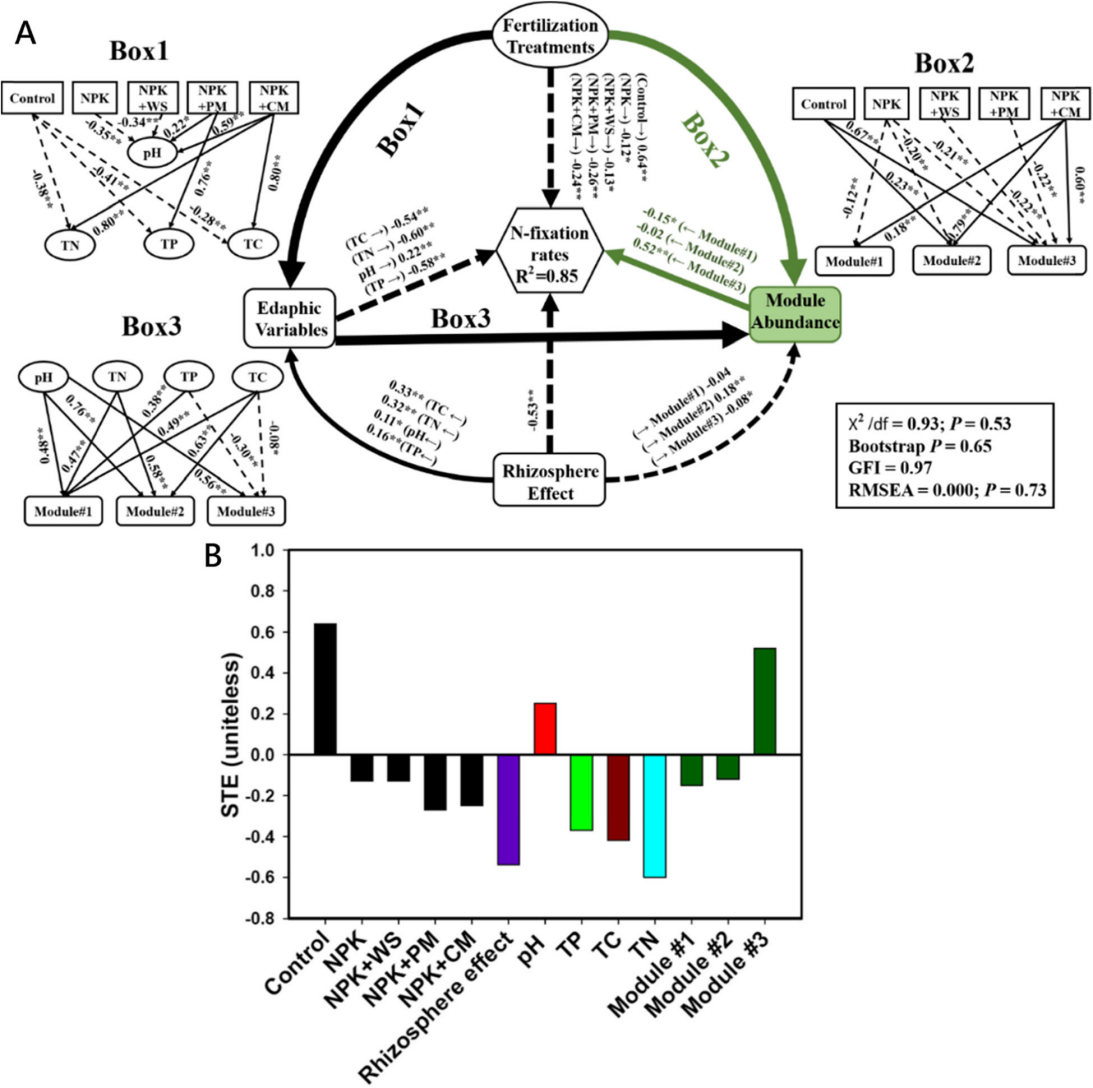
**a** A structural equation model describing the effects of relative abundance of main ecological clusters on the nitrogen fixation rates. Soil properties include soil pH, total phosphorus (TP), total carbon (TC), and total nitrogen (TN). Numbers labeling the arrow lines are indicative of the correlations. *R*^2^ denotes the proportion of variance explained. Significance levels of each predictor are *P < 0.05 and **P < 0.01. **b** Standardized total effects (STE) from the SEM. This is the sum of direct and indirect effects from each variable on nitrogen fixation rate. Fertilization treatments: control, non-fertilization; NPK fertilization, NPK (urea, superphosphate, and potassium chloride); NPK + WS, NPK with wheat straw; NPK + PM, NPK with pig manure; NPK + CM, NPK with cow manure (NPK + CM)

Structural equation modeling (SEM) was then used to evaluate the potential associations between ecological clusters of N fixers and N fixation rates under different fertilization scenarios, and to gain a deeper knowledge of the indirect and direct effects of fertilization on N fixation when considering multiple environmental factors simultaneously. Our SEM explained 85% of the variation in N fixation rates (Fig. 4a). The relative abundance of module #1 and module #2 had direct negative effects on N fixation rates. However, a positive and significant association was still found between the relative abundance of module #3 and N fixation rates (Fig. 4). Thus, it indicated that long-term fertilization indirectly reduced N fixation by decreasing the relative abundance of diazotrophs within module #3. From a management perspective, the long-term negative effects of fertilization on N fixers in the module #3 seemed to be minimized when using NPK + CM as a fertilizer (Fig. 4a; box 2).

## Discussion

Our work provides solid evidence that, after almost four decades of experiment, fertilization largely suppressed N fixation (about 50% decrease), and the relative abundance of specific N fixers (e.g., *Geobacter* spp.) that were reported to be positively associated with N fixation rates [21]. Our SEM provided further evidence that long-term fertilization indirectly reduced N fixation rates by decreasing the relative abundance of keystone and phylogenetically clustered N fixers within module #3 (those were positively associated with N fixation rates). In addition, fertilizations resulted in a change from phylogenetic clustering to phylogenetic randomness for the N fixers within module #3. These results suggest that long-term fertilization selects against N fixation and their associated N fixers. Our work has unveiled the basic mechanisms explaining the long-term effects of fertilization on N fixation and its associated microbial communities, and further suggest that the fundamental process of N fixation, and some keystone diazotrophs, will become increasingly suppressed as we continue to increase soil fertilization.

Our results identified a subset of positive (winners) and negative (losers) associations between N fixers and long-term fertilizations. For instance, long-term fertilization was positively associated with the relative abundance of modules #1 and #2 (winners under fertilization scenarios). Taxa within the dominant genera *Bradyrhizobium* and *Burkholderia* are known to be facultative N fixers [22, 23], which can consume soil resources from fertilization to support vegetative growth instead of fixing nitrogen [24]. Moreover, these genera are found within the classes *Alphaproteobacteria* and *Betaproteobacteria*, respectively, which are often classified as copiotrophs [25]. These dominant copiotrophic soil organisms may benefit from the resources coming from fertilizers and using them to support their own growth [10]. This idea is supported by the lack of correlation between the relative abundance of these taxa and N fixation rates. Our results suggest that taxa within modules #1 and #2 could benefit from long-term fertilization scenarios, providing a list of “winner” taxa.

On the other hand, long-term fertilization was negatively correlated with the relative abundance of diazotrophs within module #3 (losers under fertilization scenarios). Interestingly, fertilization also appeared to select against taxa within this ecological cluster, which were found to be phylogenetically clustered in the unfertilized field, but became phylogenetically randomness under long-term application of fertilizations. Reductions in the relative abundance of these taxa could also negatively influence N fixation rates. In fact, long-term fertilizations were found to be indirectly negatively associated with N fixation, by decreasing the relative abundance of N fixers within module #3. This indicates that taxa within module #3 will be suppressed under long-term fertilization with negative consequences for N fixation rates. In this respect, our study identified a list of “loser” taxa including *Geobacter* and *Anaeromyxobacter*, under long-term fertilization scenarios. There are a couple of explanations for this result. The genera *Geobacter* and *Anaeromyxobacter*, which belong to the class *Deltaproteobacteria*, are often classified as oligotrophic taxa [25]. Therefore, the abundance of this type of soil organism is expected to be inhibited in high-nutrient environments [26]. Moreover, taxa within this ecological cluster could have a lower capacity to downregulate N fixation, as they are outcompeted by other taxa under high N conditions but are highly competitive under low-nutrient conditions. Supporting this idea, some taxa within module #3, including *Geobacter*, have been reported to be particularly successful in fixing N in nutrient-poor environments [27].

## Conclusions

Overall, our results suggest that long-term fertilization dramatically suppressed N fixation rates and the relative abundance of keystone and phylogenetically clustered N fixers. These findings have deepened our understanding on the linkage between N fixation and its associated N fixers under long-term fertilization scenarios. Moreover, our work provide solid evidence that the fundamental process of N fixation, and its associated microbial communities, will become more and more suppressed as terrestrial fertilization continues to increase.

## Methods

### Experimental design and sample collection

The experiment was set up in 1982 in Mengcheng county, Anhui province, China (33° 13′ N, 116° 35′ E, 42 m elevation) with typical lime concretion black soil. The annual temperature is 14.8 °C and the annual precipitation is 872 mm. Five fertilization treatments with wheat-soybean crop rotation were compared in a completely randomized block design with four replicates (each plot is 70 m^2^) [28, 29]: (1) control, non-fertilization; (2) NPK, NPK chemical fertilizers comprising urea (180 kg N ha^−1^ year^−1^), superphosphate (90 kg P_2_O_5_ ha^−1^ year^−1^) and potassium chloride (86 kg K_2_O ha^− 1^ y^−1^); (3) NPK + WS, NPK chemical fertilizers plus 7500 kg wheat straw ha^−1^ year^−1^; (4) NPK + PM, NPK chemical fertilizers plus 15,000 kg fresh pig manure ha^−1^ year^−1^; (5) NPK + CM, NPK chemical fertilizers plus 30,000 kg fresh cow manure ha^−1^ year^−1^. In the NPK + WS treatment, all the wheat straw were returned to the field, the pig manure in the NPK + PM treatment and the cow manure in the NPK + CM had the similar amount of organic carbon with the added wheat straw. Moreover, these contrasting types of fertilizers included in our fertilization treatments have different levels of availability for plants and microbes, e.g., from more labile (pig manure) to more recalcitrant (wheat straw and cow manure). We used a wide range of fertilization treatments aiming to make our results representative and applicable to contrasting management practices.

We dug around the wheat group (containing 30 to 40 wheat plants during the wheat filling stage on the 20th of April, 2017) to keep the root systems as intact as possible. The rhizosphere soil that was tightly adhered to the roots was then brushed. At the same time, the topsoil (0–15 cm deep) was collected as bulk soil using an auger corer (approximately 20 cm away from the plants). The collected soil was sieved through a 2 mm mesh to remove the impurities such as roots and stones. Some of the soil was stored at 4 °C for chemical analyses, and the rest was stored at − 40 °C for DNA extraction.

### Soil physicochemical analysis

A pH meter (FE20 FiveEasy™, Mettler Toledo, Germany) was used to measure the soil pH at a soil to distilled water ratio of 1:5 (weight/volume). The soil moisture was determined gravimetrically by drying 5 g of fresh soil at approximately 105–108 °C to reach a constant weight and then calculating the weight ratio (evaporated water to dried soil). The total carbon (TC) and total nitrogen (TN) contents of the soil were determined by combustion of air-dried soil using a CNS-2000 analyzer (LECO, St. Joseph, MI, USA), after sieving the soil through a 0.15 mm mesh. The total phosphorus (TP) and total potassium (TK) contents of the soil were extracted after HF-HClO_4_ digestion and measured using the molybdenum blue method and flame spectrophotometry method (FP640, INASA, China), respectively. Dissolved organic carbon (DOC) was extracted by adding 50 mL distilled water to 5 g fresh soil, shaking for 1 h, and vacuum filtering through a G4 glass fiber filter with a pore space of 1.2 μm (Fisher), and then, the carbon contents in the extracts were determined by a total organic carbon analyzer (multi N/C 3000, Analytik Jena, Germany). Nitrate (NO_3_^−^-N), ammonium (NH_4_^+^-N), and dissolved total nitrogen (DTN) were extracted at a ratio of 5 g fresh soil to 50 mL 2 M KCl. After shacking for 1 h, the extracts were filtered through a G4 glass fiber filter with a pore space of 1.2 μm (Fisher), and then, a continuous flow analytical system (San^++^ system, Skalar, Holland) was used to analyze the content of NO_3_^−^-N, NH_4_^+^-N, and DTN. Dissolved organic nitrogen (DON) was calculated using the following formula: DON = DTN − NH_4_^+^-N − NO_3_^−^-N. The available phosphorus (AP) in the soil was extracted by 0.5 M NaHCO_3_ and determined by using molybdenum blue method. Available potassium (AK) was extracted by 1 M ammonium acetate and determined by flame photometer (FP640, INASA, China) (Additional file 3: Appendix 1).

### Determination of nitrogen fixation rates

The ^15^N_2_-labeling method is one of the most common and widely applied methods used for measuring N fixation rates [30, 31]. Five grams of soil were placed into 18 × 150 mm Balch tubes, and the headspace was replaced with synthetic air containing 80% ^15^N_2_ and 20% O_2_. The controls were filled with unlabeled N_2_ gas and processed in parallel. The tubes were incubated horizontally in the dark at room temperature for 22 days. The atom % ^15^N of soil samples was determined using a stable isotope ratio mass spectrometer (Flash 2000 HT/Conflo IV/Delta V, Thermo Fisher Scientific, Germany). Then, we calculated the net potential N fixation rate by comparing the difference of total ^15^N in soils receiving ^15^N_2_ relative to control.

### High-throughput sequencing and bioinformatics analysis

For the DNA extraction, 0.5 g of fresh soil was used with the Fast DNA SPIN Kit (MP Biomedicals, Santa Ana, CA, USA). The *nifH* genes were amplified using primer pairs *nifH*-F/*nifH*-R (5′-AAAGGYGGWATCGGYAARTCCACCAC-3′)/(5′-TTGTTSGCSGCRTACATSGCCATCAT-3′) [32]. PCR reactions were performed in a 20 μL reaction containing 4 μL of 5 × FastPfu buffer, 2 μL of 2.5 mM dNTPs, 0.8 μL of 5 μM forward primer, 0.8 μL of 5 μM reverse primer, 0.4 μL of fastPfu Polymerase, 10 ng of template DNA, and double distilled water (ddH_2_O). Amplification was performed at 95 °C for 3 min, with 35 cycles of 95 °C for 30 s, 55 °C for 30 s, and 72 °C for 45 s, and extension at 72 °C for 10 min. PCR amplicons were purified by Agarose Gel DNA purification kit (TaKaRa Bio), and triplicate PCR amplifications for each sample were conducted and pooled as a PCR product and then sequenced on the platform of Illumina MiSeq PE300 (Majorbio Company in Shanghai, China). After sequencing, the *nifH* nucleotide sequences were analyzed using the QIIME-1.9.1 pipeline (http://qiime.sourceforge.net/) [33]. Firstly, the low-quality sequences (those with a quality score < 20, containing ambiguous nucleotides, or not matching the primer and barcode) were removed and the remaining sequences were further converted to amino acid sequences using the FunGene Pipeline of the Ribosomal Database Project [34]. The sequences encoding proteins that did not match the *nifH* protein sequence or that contained termination codons were discarded. The remaining sequences were aligned against the *nifH* gene database [35], removing both the failed and chimeric sequences. The remaining high-quality sequences were clustered into operational taxonomic units (OTUs) at 95% similarity with UCLUST [36] running in de novo mode, and all singleton OTUs were deleted.

### Co-occurrence network analysis

We constructed a co-occurrence network with all the samples (rhizosphere and bulk soil) and identified the main ecological clusters of strongly associated OTUs. The top OTUs, accounting for more than 80% of the relative abundance in the total community, were chosen [37]. All pair-wised Spearman correlations between OTUs were calculated, and the correlations with a Spearman’s coefficient of less than 0.65 and a *P* value of more than 0.01, were removed. This allowed us to focus only on the OTUs that strongly co-occurred and were more likely to interact with each other. The main modules (ecological clusters) in the network were visualized using Gephi (https://gephi.org/). The relative abundance of each ecological cluster was calculated by averaging the standardized relative abundances (z-score) of the species that belonged to it (Additional file 3: Appendix 3).

### Statistical analysis

ANOVA and pairwise *t* tests were used to compare the soil variables, dominant microbial taxa, and the microbial alpha diversity between different fertilization treatments (Additional file 3: Appendix 1). These tests were implemented using SPSS 21. Mantel test was used to analyze the correlations between the diazotrophic community and physicochemical properties (Additional file 3: Appendix 2). This was performed using the “vegan” package in R × 32 (3.2.2). A principal coordinate analysis (PCoA) was used to find significant differences in diazotrophic communities between sampling groups (Additional file 3: Appendix 2). The PCoA was carried out using the “labdsv” package R × 32 (3.2.2) (http://cran.stat.sfu.ca/).

### Phylogenetic analyses

The *nifH* gene provides sufficient phylogenetic resolution [38] in ecological studies. The phylogenetic tree for the 481 dominant diazotrophic phylotypes in the ecological clusters was built using FastTree [39], and visualized using GraPhlAn [40]. Phylogenetic sampling theory can be analytically employed (assuming the random sampling from the phylogenetic tree as the predicted phylogenetic diversity in a local community and then comparing the observed phylogenetic diversity with those predictions) [20] to determine the degree to which diazotrophic community appear random (between − 2 and + 2), over-dispersed (above + 2), or clustered (below − 2). Phylogenetic sampling theory was performed using the R package “picante” [41]. An advantage of randomly sampling the regional phylogenetic tree is that it can be used to compare samples of unequal sizes based on the binomial sampling model [42]. The differences between observed and expected phylogenetic diversity were determined by calculating and comparing z-scores for each ecological cluster. When the observed phylogenetic diversity is less than the expected diversity (below − 2), the microbial community in the ecological cluster is considered to be phylogenetically clustered, which means that closely related taxa are more likely to be sampled [43] and actively selected by the environment [42].

### Structural equation modeling analysis

The SEM [44] was conducted using IBM SPSS Amos 21 (Chicago, IL: Amos Development Corporation). It was used to evaluate the direct and indirect effects of the soil physicochemical properties and the relative abundance of the main ecological clusters on the N fixation rates. The physiochemical properties of the soil included soil pH, total carbon, total nitrogen, and total phosphorus. In the model, the treatments (control, NPK, NPK + WS, NPK + PM, and NPK + CM) were categorical variables with two levels: 1 (a particular treatment) and 0 (remaining considered treatments). In addition, bootstrapping was used to test the probability that path coefficients differed from zero, as a few of the variables were not normally distributed. We also calculated the standardized total effects (STEs) of the soil properties, fertilization treatments, and rhizosphere effect on the N fixation rate to aid interpretation of the SEM.

### Random Forest modeling analysis

Random Forest regression (R package “randomForest”) was used to regress the normalized OTUs in different treatments. The 10-fold cross-validation method was used to determine the optimal set of OTUs correlated to the N fixation rates [45]. Ranked lists of OTUs in order of Random Forests reported feature importance scores were achieved based on the increase in mean-square error of nitrogen fixation rates predicted over 100 iterations of the algorithm. The 50 marker OTUs were chosen based on the minimum average cross-validation mean-squared errors, which were obtained from five trials of the 10-fold cross-validation.

## Supplementary information

**Additional file 1:** **Table S1.** Physicochemical soil properties in bulk soil and rhizosphere soil among different fertilization treatments. **Table S2.** The relative abundance of dominant diazotrophic genera among different treatments. **Table S3.** Pairwise t-tests for the relative abundance of dominant diazotrophic genera between bulk soil and rhizosphere soil in different fertilization treatments. **Table S4.** Comparison of diazotrophic alpha-diversity indexes among different fertilization treatments. **Table S5.** Pairwise t-testing of diazotrophic alpha-diversity in bulk soil and rhizosphere soil. **Table S6.** Pairwise t-tests of nitrogen fixation rates in bulk soil and rhizosphere soil in different fertilization treatments. **Table S7.** ADONIS double factor analysis for the diazotrophic community. **Table S8.** Pairwise t-tests of relative abundance of main ecological clusters within the diazotrophic community in bulk soil and rhizosphere soil under different fertilization treatments. **Table S9.** The network properties for the main ecological clusters of the diazotrophic community. **Table S10.** Operational taxonomic unit (OTU) count properties of important species for nitrogen fixation rates found by the Random Forest model in the three main ecological clusters. **Table S11.** The correlations (r) and significance (p) were determined using a Mantel test between the diazotrophic community and environmental variables in bulk soil and rhizosphere soil. **Table S12.** Spearman correlation between physicochemical soil properties and diazotrophic alpha-diversity. **Table S13.** Spearman correlation between physicochemical soil properties and relative abundance of the main diazotrophic ecological clusters.

**Additional file 2:** **Figure S1.** Relative abundance of the dominant diazotrophic genera in different fertilization treatments. **Figure S2.** A random forest model was applied to regress the diazotrophic OTU profiling in bulk soil and rhizosphere soil against the nitrogen fixation rates. **Figure S3.** Correlations between the relative abundance of important species for nitrogen fixation rates found by the Random Forest model and their Importance Index in different fertilization treatments. **Figure S4.** Diazotrophic community variations in different fertilization samples; and diazotrophic community composition variations which were based on Bray-Curtis distances by principal coordinate analysis.

**Additional file 3.** Supplementary Results. **Appendix 1.** Soil properties and diazotrophic community under long-term fertilization scenarios. **Appendix 2.** Edaphic factors associated with the soil diazotrophic community under long-term fertilization scenarios. **Appendix 3.** Diazotrophic ecological clusters and associated edaphic factors.

## Data Availability

The obtained sequences were submitted to the NCBI Sequence Read Archive (SRA) with accession number SRP149667 (https://www.ncbi.nlm.nih.gov/sra/SRP149667). Other data and result supporting the findings of the study are available in this article and its supplementary information files.
