# Supplementary material for: Suppressed N fixation and diazotrophs after four decades of fertilization
Source: Microbiome. 2019 Oct 31;7:143. doi: 10.1186/s40168-019-0757-8 (PMC6824023; doi:10.1186/s40168-019-0757-8)
Supplement: Supplementary file 1 — Additional file 1: Table S1. Physicochemical soil properties in bulk soil and rhizosphere soil among different fertilization treatments. Table S2. The relative abundance of dominant diazotrophic genera among different treatments. Table S3. Pairwise t-tests for the relative abundance of dominant diazotrophic genera between bulk soil and rhizosphere soil in different fertilization treatments. Table S4. Comparison of diazotrophic alpha-diversity indexes among different fertilization treatments. Table S5. Pairwise t-testing of diazotrophic alpha-diversity in bulk soil and rhizosphere soil. Table S6. Pairwise t-tests of nitrogen fixation rates in bulk soil and rhizosphere soil in different fertilization treatments. Table S7. ADONIS double factor analysis for the diazotrophic community. Table S8. Pairwise t-tests of relative abundance of main ecological clusters within the diazotrophic community in bulk soil and rhizosphere soil under different fertilization treatments. Table S9. The network properties for the main ecological clusters of the diazotrophic community. Table S10. Operational taxonomic unit (OTU) count properties of important species for nitrogen fixation rates found by the Random Forest model in the three main ecological clusters. Table S11. The correlations (r) and significance (p) were determined using a Mantel test between the diazotrophic community and environmental variables in bulk soil and rhizosphere soil. Table S12. Spearman correlation between physicochemical soil properties and diazotrophic alpha-diversity. Table S13. Spearman correlation between physicochemical soil properties and relative abundance of the main diazotrophic ecological clusters. [file 40168_2019_757_MOESM1_ESM.zip › Fan et al._Supplementary Tables.docx]

**Table S1. Physicochemical soil properties in bulk soil and rhizosphere soil among different fertilization treatments.** *Values are means (SD). Values in the same row followed by different letters (a, b, c) differed significantly at P < 0.05 (Duncan’s test). BS: Bulk soil; RS: Rhizosphere soil. Fertilization treatments: Control, non-fertilization; NPK fertilization, NPK (urea, superphosphate, and potassium chloride); NPK+WS, NPK with wheat straw; NPK+PM, NPK with pig manure; NPK+CM, NPK with cow manure. DTN: dissolved total nitrogen; NO_3_^-^-N: nitrate nitrogen; NH_4_^+^-N: ammonium nitrogen; DON: dissolved organic nitrogen; DOC: dissolved organic carbon; TC: total carbon; TN: total nitrogen; TP: total phosphorus; TK: total potassium; AP: available phosphorus; AK: available potassium.

Please see the excel file (SI_Table S1.xlsx).

**Table S2. The relative abundance of dominant diazotrophic genera among different treatments**. Values in the same row followed by different letters (a, b, c) differed significantly at P < 0.05 (Duncan’s test). For abbreviations see Table S1.

| **Genus level** | **Bulk Soil** | | | | | | | | | | |  |
| --- | --- | --- | --- | --- | --- | --- | --- | --- | --- | --- | --- | --- |
|  | **Control** | | | **NPK** | **NPK+WS** | | **NPK+PM** | | | **NPK+CM** | |  |
| **Bradyrhizobium** | 0.19(0.14)b | | | 0.6(0.12)a | 0.61(0.17)a | | 0.61(0.11)a | | | 0.32(0.03)b | | |
| **Burkholderia** | 0.19(0.05)a | | | 0.008(0.003)b | 0.006(0.005)b | | 0.12(0.09)a | | | 0.19(0.03)a | | |
| **Geobacter** | 0.06(0.04)b | | | 0.12(0.04)a | 0.17(0.1)a | | 0.04(0.01)b | | | 0.09(0.03)ab | | |
| **Polaromonas** | 0.12(0.08)a | | | 0.01(0.01)b | 0.003(0.002)b | | 0.04(0.02)b | | | 0.11(0.01)a | | |
| **Xanthobacter** | 0.002(0.001)b | | | 0.088(0.06)a | 0.035(0.01)b | | 0.005(0.003)b | | | 0.004(0.001)b | | |
| **Dechloromonas** | 0.012(0.007)a | | | 0.03(0.01)a | 0.011(0.01)a | | 0.027(0.002)a | | | 0.034(0.003)a | | |
| **Anaeromyxobacter** | 0.008(0.006)b | | | 0.05(0.02)a | 0.08(0.04)a | | 0.007(0.001)b | | | 0.0034(0.001)b | | |
| **Rhizobium** | 0.02(0.01)b | | | 0.009(0.001)b | 0.004(0.002)b | | 0.012(0.002)b | | | 0.05(0.01)a | | |
| **Leptothrix** | 0.03(0.01)a | | | 0.005(0.002)b | 0.005(0.002)b | | 0.012(0.01)b | | | 0.012(0.002)b | | |
| **Dechlorosoma** | 0.011(0.01)b | | | 0.003(0.002)b | 0.0024(0.001)b | | 0.05(0.02)a | | | 0.007(0.003)b | | |
| **Hyphomicrobium** | 0.02(0.01)a | | | 0.006(0.002)c | 0.007(0.002)c | | 0.01(0.01)b | | | 0.016(0.01)ab | | |
| **Others** | 0.34(0.01)a | | | 0.06(0.02)c | 0.06(0.02)c | | 0.08(0.02)c | | | 0.16(0.04)b | | |
| **Genus level** | **Rhizosphere Soil** | | | | | | | | | | | |
|  | **Control** | **NPK** | | | **NPK+WS** | | **NPK+PM** | | **NPK+CM** | | | |
| **Bradyrhizobium** | 0.15(0.09)b | | 0.76(0.07)a | | | 0.77(0.09)a | | 0.55(0.12)a | | | 0.27(0.08)b | |
| **Burkholderia** | 0.23(0.02)b | | 0.022(0.018)c | | | 0.005(0.002)c | | 0.15(0.08)b | | | 0.33(0.07)a | |
| **Geobacter** | 0.077(0.05)a | | 0.026(0.008)a | | | 0.11(0.05)a | | 0.012(0.01)a | | | 0.015(0.005)a | |
| **Polaromonas** | 0.16(0.03)a | | 0.0051(0.003)c | | | 0.0013(0.0007)d | | 0.052(0.04)d | | | 0.1(0.03)b | |
| **Xanthobacter** | 0.003(0.0009)b | | 0.045(0.004)a | | | 0.027(0.005)a | | 0.0058(0.006)b | | | 0.0026(0.002)b | |
| **Dechloromonas** | 0.02(0.01)a | | 0.006(0.004)b | | | 0.006(0.005)b | | 0.06(0.05)a | | | 0.005(0.003)b | |
| **Anaeromyxobacter** | 0.007(0.006)b | | 0.013(0.01)a | | | 0.015(0.01)a | | 0.005(0.002)b | | | 0.003(0.002)b | |
| **Rhizobium** | 0.03(0.01)a | | 0.009(0.001)b | | | 0.002(0.001)b | | 0.007(0.001)b | | | 0.021(0.002)a | |
| **Leptothrix** | 0.049(0.02)a | | 0.0046(0.002)c | | | 0.0044(0.002)c | | 0.019(0.003)b | | | 0.017(0.005)b | |
| **Dechlorosoma** | 0.014(0.02)a | | 0.0002(0.0001)b | | | 0.0002(0.0001)b | | 0.043(0.002)a | | | 0.0026(0.003)a | |
| **Hyphomicrobium** | 0.023(0.02)a | | 0.0065(0.003)b | | | 0.0065(0.002)b | | 0.013(0.01)a | | | 0.017(0.01)a | |
| **Others** | 0.24(0.01)a | | 0.103(0.03)b | | | 0.056(0.02)b | | 0.09(0.05)b | | | 0.22(0.07)a | |

**Table S3. Pairwise t-tests for the relative abundance of dominant diazotrophic genera between bulk soil and rhizosphere soil in different fertilization treatments.** Bold values indicate a significant difference between bulk soil and rhizosphere soil. For abbreviations see Table S1.

Please see the excel file (SI_Table S3.xlsx).

**Table S4. Comparison of diazotrophic alpha-diversity indexes among different fertilization treatments**. Values are means (SD). Values in the same row followed by different letters differed significantly at P < 0.05 (Duncan’s test). For abbreviations see Table S1.

| **Alpha-diversity** | **Bulk Soil** | | | | |
| --- | --- | --- | --- | --- | --- |
|  | **Control** | **NPK** | **NPK+WS** | **NPK+PM** | **NPK+CM** |
| **Richness** | 920(277)a | 395(76)c | 360(92)c | 611(138)b | 875(118)a |
| **Phylogenetic Diversity** | 262(39)a | 181(20)c | 167(21)c | 216(27)b | 261(20)a |
| **Alpha-diversity** | **Rhizosphere Soil** | | | | |
|  | **Control** | **NPK** | **NPK+WS** | **NPK+PM** | **NPK+CM** |
| **Richness** | 989(134)a | 236(45)c | 188(81)c | 594(251)b | 725(92)b |
| **Phylogenetic Diversity** | 270(24)a | 137(17)c | 113(23)c | 199(50)b | 227(16)ab |

**Table S5. Pairwise t-testing of diazotrophic alpha-diversity in bulk soil and rhizosphere soil.** Bold values indicate a significant difference between bulk soil and rhizosphere soil. For abbreviations see Table S1.

| **Alpha-diversity** | **BS (Mean)** | **RS (Mean)** | **BS (SD)** | **RS (SD)** | **t stat** | **P-value** |
| --- | --- | --- | --- | --- | --- | --- |
| **Richness** | 632 | 546 | 277 | 333 | 2.57 | **0.019** |
| **Phylogenetic Diversity** | 218 | 189 | 46 | 64 | 4.05 | **0.001** |

**Table S6. Pairwise t-tests of nitrogen fixation rates in bulk soil and rhizosphere soil in different fertilization treatments**. Bold values indicate the significant difference between bulk soil and rhizosphere soil. For abbreviations see Table S1.

| **Treatments** | **BS (Mean)** | **RS (Mean)** | **BS (SD)** | **RS (SD)** | **t stat** | **P-value** |
| --- | --- | --- | --- | --- | --- | --- |
| **Control** | 514.9 | 306.1 | 20.4 | 20.7 | 5.48 | **0.012** |
| **NPK** | 295.1 | 170.4 | 19.4 | 23.2 | 9.24 | **0.003** |
| **NPK+WS** | 246.3 | 215.9 | 17.6 | 28.9 | 1.49 | 0.09 |
| **NPK+PM** | 224.2 | 175.5 | 17.9 | 36.2 | 3.74 | **0.05** |
| **NPK+CM** | 254.7 | 153.9 | 22.4 | 10.1 | 9.71 | **0.002** |

**Table S7. ADONIS double factor analysis for the diazotrophic community**. For abbreviations see Table S1.

| **ADONIS double factor analysis** | | | | | |
| --- | --- | --- | --- | --- | --- |
| **Between Treatments** | | | **Between BS and RS** | | |
| **F** | **R** | **P** | **F** | **R** | **P** |
| 16.9 | 0.65 | 0.001 | 2.0 | 0.02 | 0.09 |

**Table S8. Pairwise t-tests of relative abundance of main ecological clusters within the diazotrophic community in bulk soil and rhizosphere soil under different fertilization treatments**. Bold values indicate a significant difference between bulk soil and rhizosphere soil. For abbreviations see Table S1.

| **Ecological Clusters** | **BS (Mean)** | | **RS (Mean)** | **BS (SD)** | **RS (SD)** | | **t stat** | **P-value** |
| --- | --- | --- | --- | --- | --- | --- | --- | --- |
| **Module #1** | 0.44 | 0.41 | | 0.09 | 0.14 | 1.17 | | 0.26 |
| **Module #2** | 0.22 | 0.25 | | 0.06 | 0.07 | -1.81 | | 0.08 |
| **Module #3** | 0.23 | 0.22 | | 0.08 | 0.07 | 0.59 | | 0.56 |

**Table S9. The network properties for the main ecological clusters of the diazotrophic community**.

| **Ecological Clusters** | **Nodes** | | **Edges** | **Density** | **Negative correlation** | **Transitivity** |
| --- | --- | --- | --- | --- | --- | --- |
| **Module #1** | 150 | 6281 | | 0.28 | 0 | 0.78 |
| **Module #2** | 166 | 7663 | | 0.28 | 0 | 0.83 |
| **Module #3** | 189 | 7508 | | 0.21 | 36.5% | 0.69 |

**Table S10. Operational taxonomic unit (OTU) count properties of important species for nitrogen fixation rates found by the Random Forest model in the three main ecological clusters**.

| **Random Forest** | **Treatments** | **Module#1** | **Module#2** | **Module#3** |
| --- | --- | --- | --- | --- |
| **N-fixation rates**  **(50)** | **Control** | 6 | 6 | 15 |
|  | **NPK** | 1 | 3 | 27 |
|  | **NPK+WS** | 1 | 2 | 20 |
|  | **NPK+PM** | 3 | 5 | 19 |
|  | **NPK+CM** | 1 | 6 | 17 |
|  | **Average** | 3 | 4 | 20 |

**Table S11. The correlations (r) and significance (p) were determined using a Mantel test between the diazotrophic community and environmental variables in bulk soil and rhizosphere soil.**

| **Group** | **ALL** | **BS** | **RS** |
| --- | --- | --- | --- |
| **Moisture** | 0.117 (0.021) | 0.18（0.062） | 0.03（0.34） |
| **pH** | 0.733 (0.001) | 0.73（0.001） | 0.76（0.001） |
| **NO_3_^-^-N** | 0.024 (0.306) | 0.36（0.002） | -0.06（0.75） |
| **NH_4_^+^-N** | 0.093 (0.074) | 0.216（0.031） | -0.004（0.52） |
| **DON** | 0.096 (0.074) | 0.131（0.102） | 0.032（0.35） |
| **DOC** | 0.208 (0.001) | 0.155（0.047） | 0.331（0.002） |
| **TC** | 0.29 (0.001) | 0.325（0.004） | 0.27（0.01） |
| **TN** | 0.457 (0.001) | 0.459（0.001） | 0.48（0.001） |
| **TP** | 0.344 (0.001) | 0.279（0.008） | 0.336（0.004） |
| **TK** | 0.093 (0.071) | 0.265（0.014） | -0.013（0.55） |
| **AP** | 0.24 (0.002) | 0.186（0.036） | 0.198（0.025） |
| **AK** | 0.188 (0.008) | 0.243（0.012） | 0.21（0.014） |

**Table S12. Spearman correlation between physicochemical soil properties and diazotrophic alpha-diversity**. For abbreviations see Table S1.

| **Spearman Correlation** | **Richness** | **PD** |
| --- | --- | --- |
| **Moisture** | 0.30 | 0.32* |
| **pH** | 0.79** | 0.77** |
| **NO_3_^-^-N** | -0.08 | -0.07 |
| **NH_4_^+^-N** | -0.52** | -0.53** |
| **DON** | -0.59** | -0.61** |
| **DOC** | -0.35* | -0.39* |
| **TC** | -0.08 | -0.11 |
| **TN** | -0.11 | -0.13 |
| **TP** | -0.14 | -0.15 |
| **TK** | 0.099 | 0.11 |
| **AP** | -0.09 | -0.08 |
| **AK** | -0.06 | -0.08 |

**Table S13. Spearman correlation between physicochemical soil properties and relative abundance of the main diazotrophic ecological clusters.** For abbreviations see Table S1.

| **Spearman Correlation** | **Module#1** | **Module#2** | **Module#3** |
| --- | --- | --- | --- |
| **Moisture** | 0.43** | 0.35* | 0.09 |
| **pH** | 0.41** | 0.78** | 0.55** |
| **NO_3_^-^-N** | 0.23 | 0.14 | -0.11 |
| **NH_4_^+^-N** | -0.16 | -0.27 | -0.28 |
| **DON** | -0.17 | -0.19 | -0.41** |
| **DOC** | 0.19 | 0.13 | -0.34* |
| **TC** | 0.39* | 0.38* | -0.22 |
| **TN** | 0.37* | 0.36* | -0.21 |
| **TP** | 0.29 | 0.21 | -0.40** |
| **TK** | 0.27 | 0.44** | -0.05 |
| **AP** | 0.31 | 0.22 | -0.46** |
| **AK** | 0.37* | 0.42** | -0.16 |
