# Supplementary material for: Suppressed N fixation and diazotrophs after four decades of fertilization
Source: Microbiome. 2019 Oct 31;7:143. doi: 10.1186/s40168-019-0757-8 (PMC6824023; doi:10.1186/s40168-019-0757-8)
Supplement: Supplementary file 2 — Additional file 2: Figure S1. Relative abundance of the dominant diazotrophic genera in different fertilization treatments. Figure S2. A random forest model was applied to regress the diazotrophic OTU profiling in bulk soil and rhizosphere soil against the nitrogen fixation rates. Figure S3. Correlations between the relative abundance of important species for nitrogen fixation rates found by the Random Forest model and their Importance Index in different fertilization treatments. Figure S4. Diazotrophic community variations in different fertilization samples; and diazotrophic community composition variations which were based on Bray-Curtis distances by principal coordinate analysis. [file 40168_2019_757_MOESM2_ESM.docx]

**
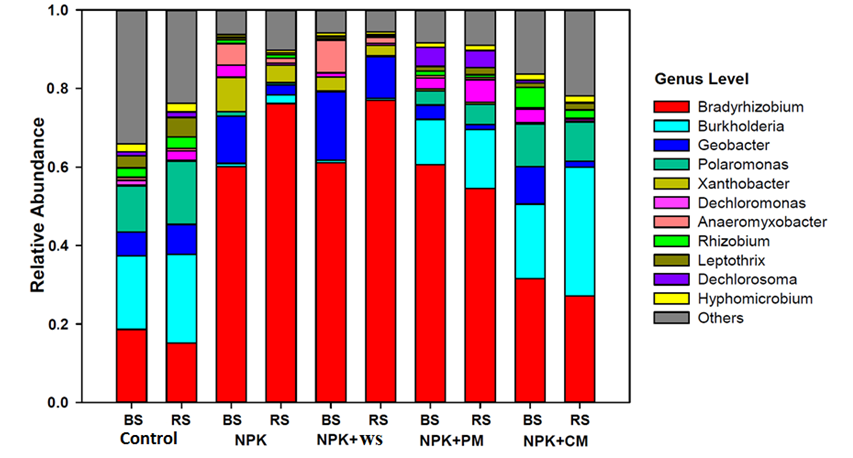
**

**Fig S1.** **Relative abundance of** **the dominant diazotrophic genera in different fertilization treatments.** BS: Bulk soil; RS: Rhizosphere soil. Control: Non-fertilization; NPK: NPK (urea, superphosphate and potassium chloride) fertilization; NPK+WS: NPK with wheat straw; NPK+PM: NPK with pig manure; NPK+CM: NPK with cow manure.

**
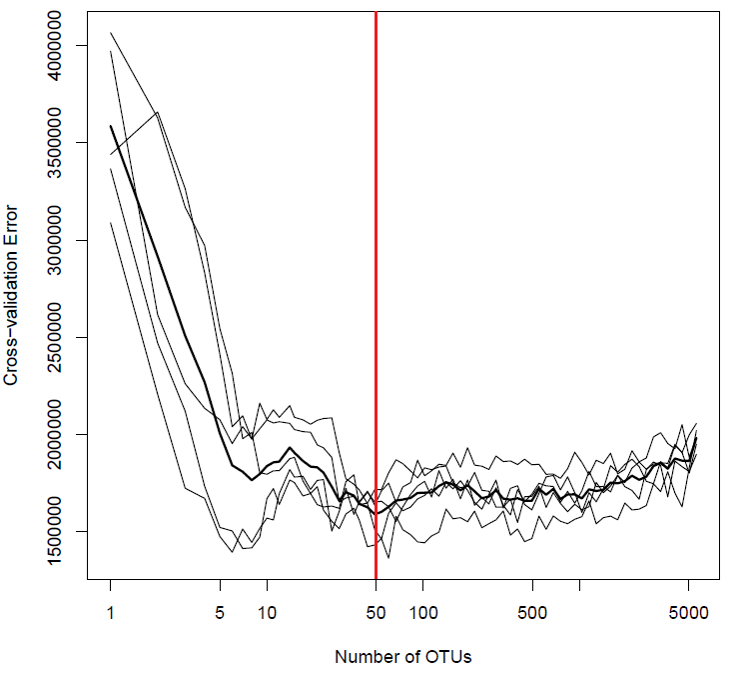
**

**Fig S2. A random forest model was applied to regress the diazotrophic OTU profiling in bulk soil and rhizosphere soil against the nitrogen fixation rates.** Five repeats of 10-fold cross-validation in the training set resulted in selection of 50 OTU markers for predicting N fixation rates.


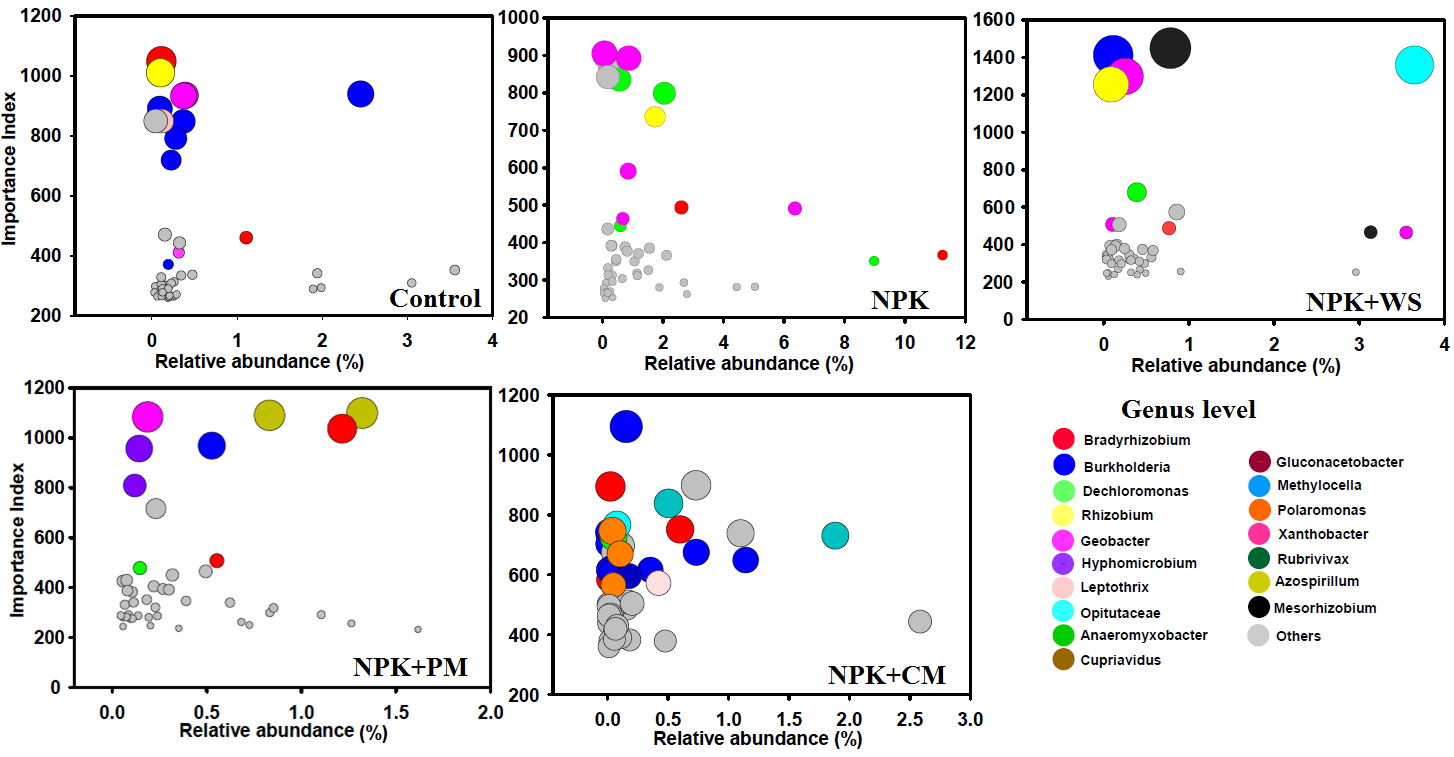


**Fig S3. Correlations between the relative abundance of important species for nitrogen fixation rates found by the Random Forest model and their Importance Index in different fertilization treatments.** (Importance Index: IncNodePurity). For abbreviations see Fig S1.

**
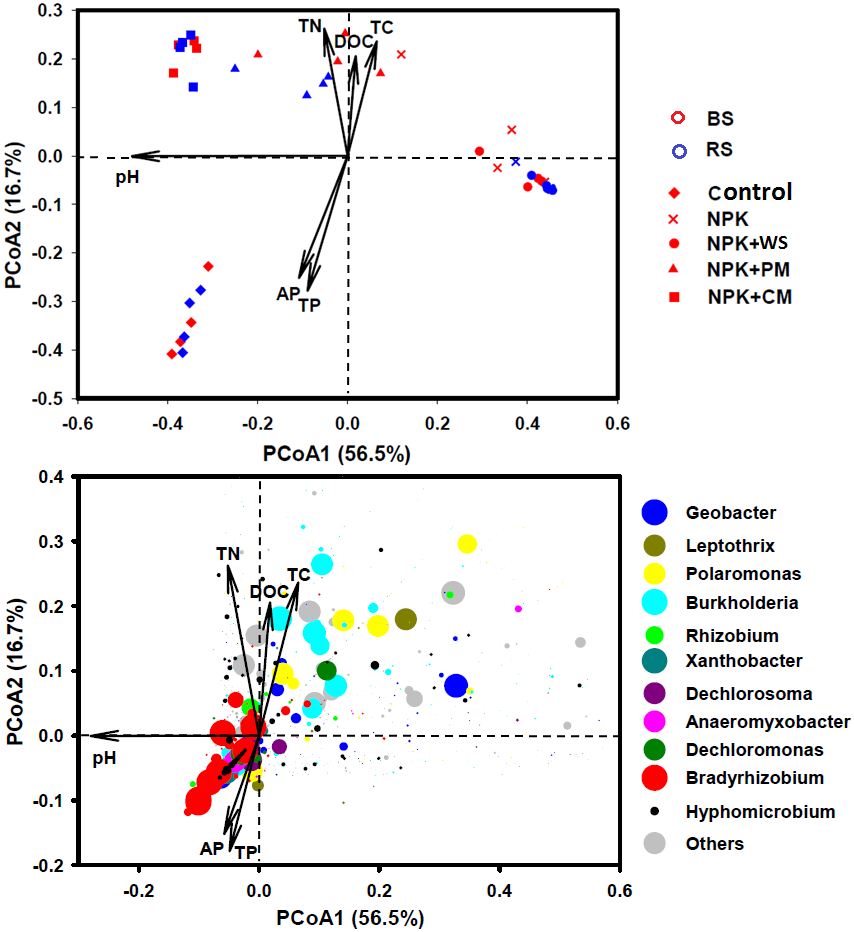
**

**Fig S4. Diazotrophic community variations in different fertilization samples; and diazotrophic community composition variations which were based on Bray-Curtis distances by principal coordinate analysis.** Circle sizes correspond to the relative abundance of diazotrophic OTUs, and colors are assigned to different genus. DOC: dissolved organic carbon; TC: total carbon; TN: total nitrogen; TP: total phosphorus; AP: available phosphorus. For abbreviations see Fig S1.
