# Supplementary material for: Suppressed N fixation and diazotrophs after four decades of fertilization
Source: Microbiome. 2019 Oct 31;7:143. doi: 10.1186/s40168-019-0757-8 (PMC6824023; doi:10.1186/s40168-019-0757-8)
Supplement: Supplementary file 3 — Additional file 3. Supplementary Results. Appendix 1. Soil properties and diazotrophic community under long-term fertilization scenarios. Appendix 2. Edaphic factors associated with the soil diazotrophic community under long-term fertilization scenarios. Appendix 3. Diazotrophic ecological clusters and associated edaphic factors. [file 40168_2019_757_MOESM3_ESM.docx]

**Supplementary Results**

**Appendix 1. Soil properties and diazotrophic community under long-term fertilization scenarios**

The long-term application of NPK chemical fertilizers significantly decreased the soil pH and increased the contents of most soil nutrients (such as total nitrogen, total phosphorus and total potassium). Meanwhile the addition of organic manures (especially cow manure) significantly recovered the soil pH and further increased the content of soil total carbon, total nitrogen, total potassium, total phosphorus and available phosphorus (Table S1).

Thirty-five years of applying NPK chemical fertilizers significantly increased the relative abundance of *Bradyrhizobium* and decreased the relative abundance of *Burkholderia* and *Polaromonas* in both the rhizosphere soil and bulk soil (Fig S1; Table S2). However, the addition of pig manure and cow manure presented the opposite pattern. Of note, the addition of pig manure significantly increased the relative abundance of *Dechlorosoma* and *Dechloromonas*, and the addition of cow manure significantly increased the relative abundance of *Rhizobium* (Table S2). Meanwhile, *Bradyrhizobium, Burkholderia* and *Dechloromonas* were highly enriched in the rhizosphere soil, while *Geobacter* was significantly depleted in the rhizosphere soil (Table S3). Further, long-term application of NPK fertilization significantly decreased the diazotrophic diversity in both the bulk soil and rhizosphere soil; the addition of pig and cow manure significantly recovered the diazotrophic diversity (Table S4). In general, diazotrophic diversity was lower in the rhizosphere soil when compared with that in bulk soil (Table S5). Adonis analysis showed no significant variation in the diazotrophic community between bulk soil and rhizosphere soil, but found significant variation across the different fertilization treatments (Table S7).

**Appendix 2. Edaphic factors associated with the soil diazotrophic community under long-term fertilization scenarios**

A Mantel test showed that soil pH was one of the most important variables in affecting the diazotrophic community in both bulk soil and rhizosphere soil (Table S11). Meanwhile, total carbon, total nitrogen, and total phosphorus also significantly correlated with the diazotrophic community (Table S11). Principle coordinate analysis showed that soil pH, total and available phosphorus, total nitrogen, total carbon, and dissolved organic carbon were the key edaphic factors affecting the diazotrophic community structure in different fertilization treatments (Fig S4). Spearman correlation showed that diazotrophic diversity positively correlated with soil pH, while negatively correlated with ammonium nitrogen, dissolved organic nitrogen, and dissolved organic carbon (Table S12).

**Appendix 3. Diazotrophic ecological clusters and associated edaphic factors**

We found three main diazotrophic ecological clusters. The density of Module #3 (0.21) was lower than that of Module #1 (0.28) and #2 (0.28). Module #3 had more negative correlations (36.5 %) when compared with Module #1 and Module #2 (Table S9). This suggests a relatively fiercer competition pattern in Module #3. In addition, soil pH significantly correlated with the relative abundance of these three main ecological clusters (Module #1, Module #2, and Module # 3). Soil moisture, total carbon and total nitrogen significantly correlated with the relative abundance of Module #1 and Module #2; total phosphorus, available phosphorus, dissolved organic nitrogen, and dissolved organic carbon were significantly negatively correlated with relative abundance of Module #3 (Table S13).
